# Systemic Glutathione as a Skin-Whitening Agent in Adult

**DOI:** 10.1155/2020/8547960

**Published:** 2020-04-24

**Authors:** I. B. S. Sitohang, S. Ninditya

**Affiliations:** ^1^Division of Cosmetic Dermatology, Department of Dermatology and Venereology, Faculty of Medicine, Universitas Indonesia, Dr. Cipto Mangunkusumo Hospital, Jakarta, Indonesia; ^2^Faculty of Medicine, Universitas Indonesia, Jakarta, Indonesia

## Abstract

**Objectives:**

To compare the efficacy and safety profiles of systemic glutathione as a skin-whitening agent in adults from several randomized controlled trials (RCTs).

**Methods:**

This study is an evidence-based case report with literature search conducted on Clinical Key, Cochrane, Journal of the American Academy of Dermatology, Taylor and Francis Online, ScienceDirect, and PubMed databases. Three relevant RCTs were extracted and assessed for validity, importance, and applicability.

**Results:**

From 3 included trials, one of the studies opposed glutathione as a skin-whitening agent. However, the other two showed significant results only to some parts of the body or to certain age groups. As a skin-whitening agent, studies showed that glutathione yielded other cosmetic benefits as it may improve skin elasticity and reduce skin wrinkles. Furthermore, glutathione was well tolerated in oral preparations, but not in parenteral preparations.

**Conclusions:**

Highest-evidence literatures showed that glutathione is not beneficial enough as a skin-whitening agent as it was only effective in some parts of the body and did not elicit long-lasting effects. However, its safety profiles in oral preparations were well tolerated. More researches regarding the time needed for skin color to return to its original state following drug withdrawal need to be conducted as it is yet to be discovered.

## 1. Introduction

The role of skin color in daily life is important, especially for women, as it may become one's charm. Its sociopsychological significance may exceed its biological function, even to the extent of causing cosmetic problems, resulting in lower quality of life and one's self-esteem. White-skinned individuals tan their skin, while dark-skinned individuals seek various ways to brighten their skin. Skin-whitening cosmetics are high in demand throughout Asia [1, 2].

Nowadays, skin-whitening agents, either in topical, oral, or intravenous preparations, are widely available in markets. Glutathione, one of the skin-whitening agents in cosmetic industries [1], is an antioxidant commonly found in the human body [2]. Reduced glutathione (GSH) yields several systemic effects, including improvements of liver abnormalities and diabetic complications, protection from viral infections, and antitumor activities [3]. Several in vitro experiments demonstrated that glutathione showed antimelanogenic effects; thus, it is associated with melanin production [2]. It is known that glutathione may promote pheomelanin synthesis, inhibit intracellular melanogenic enzymes, and demonstrate antioxidative as well as antiaging effects [2, 3].

Despite the facts that glutathione is widely available, the efficacy and safety profiles of systemic glutathione have yet to be fully understood. Therefore, this systematic review aims to investigate the efficacy of systemic glutathione as a skin-whitening agent in adults.

## 2. Clinical Question

Is systemic glutathione, as compared to placebo, effective as a skin-whitening agent?

## 3. Methods

### 3.1. Search Strategy

In order to answer the clinical question, literature search on Clinical Key, Cochrane, Journal of the American Academy of Dermatology, Taylor and Francis Online, Pubmed, and ScienceDirect databases on June 3^rd^, 2018, was conducted. Details on literature search are shown in Table 1.

### 3.2. Study Selection

**Figure 1:**
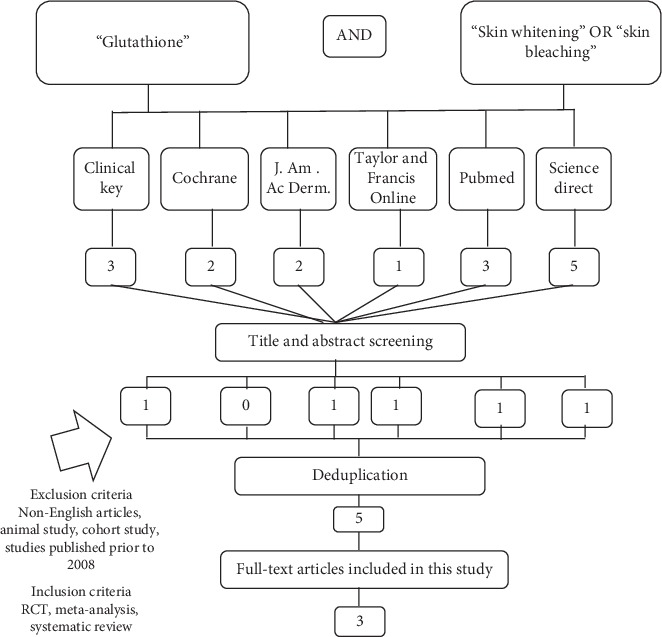
Literature search strategy.

Inclusion criteria were set to cohort studies, RCTs, meta-analyses, and systematic reviews. Conversely, exclusion criteria were also set: (1) incompatible language (articles not in English), (2) animal study, and (3) publications prior to 2008. From 16 studies, title and abstract screening followed by deduplication of similar articles were done. At the end, 3 RCTs based on validity, importance, and applicability (VIA) were included. The literature search strategy is shown in Figure 1.

## 4. Critical Appraisal

Critical appraisal of the RCTs is shown in Table 2.

## 5. Results

Based on literature search, three RCTs (Arjinpathana and Asawanonda, Zubair et al., and Weschawalit et al.) were included. This evidence-based critical appraisal will investigate the efficacy of glutathione as a skin-whitening agent (Table 3).

## 6. Discussion

Glutathione, a small, water-soluble thiol-tripeptide with low-molecular weight, is made from three amino acids: glutamate, cysteine, and glycine [5]. Glutathione is commonly found in two forms: reduced glutathione (GSH) and oxidized glutathione (GSSH). Its biological function serves as a potent antioxidant in human body [6].

Melanin, a skin pigment, consists of blackish-brown eumelanin and reddish-yellow pheomelanin. Higher pheomelanin proportion will make skin brighter [7]. Hyperpigmentation is caused by the exposure to ultraviolet radiation, resulting in the formation of reactive oxygen and nitrogen species between cells [8, 9]. Oral antioxidants will reduce melanogenesis by suppressing those free radicals [6].

Glutathione may affect skin pigmentation by inhibiting tyrosinase activity during melanogenesis, either directly or indirectly. Direct inactivation is done by binding to the active site of enzymes containing copper ion, while indirect inactivation eliminates free radicals and peroxides in antioxidative manners. During melanogenesis, glutathione converts eumelanin to pheomelanin and modulates depigmentation of melanocytotoxic agents [6].

Literature search on search engines revealed scarce results regarding this topic. Furthermore, most of the articles on this topic were published in the last 6 years, emphasizing the fact that glutathione use as a skin-whitening agent is still rare and is yet to be researched extensively on human skin.

From three appraised RCTs, two out of three demonstrated the positive effects of glutathione as a skin-whitening agent. However, Zubair et al. opposed this finding. Theoretically, glutathione level in serum will be higher after intravenous administration, thus resulting in higher adverse effects. Furthermore, long-term use of glutathione as a skin-whitening agent is not yet known [4].

The effects of glutathione on Zubair et al.'s study was lower than in the other study. This finding was suspected to arise from different genetic backgrounds of study population, degree of sun exposure, and/or different measurement scales [4]. Hong et al. demonstrated that some of the GSH content was oxidized readily and removed from circulation with a half-time of 10 minutes following an intravenous injection, and the remainder was split to three amino acid components [10].

Arjinpathana and Asawanonda showed that glutathione is effective in most sun-exposed areas of the body. This finding corroborated the hypothesis that glutathione is effective only to new melanogenesis and not to existing pigments. This is slightly different from Handog et al. who demonstrated that melanin index decreased significantly in either sun-exposed or sun-protected areas of the body. GSH acts as an antimelanogenic agent by converting eumelanin to pheomelanin systemically, with or without sun exposure [11].

Weschawalit et al. reported that both glutathione forms, GSH and GSSG, may improve skin elasticity in either sun-exposed or sun-protected areas. GSH is superior to placebo in reducing skin wrinkles, at least in some anatomical locations. GSSG and GSH showed significant effects on melanin index only in some skin areas and certain age groups, especially in the sun-exposed area of the right forearm and the GSH group with age >40 years (*p*=0.031) [3].

Campione et al. demonstrated that enzyme glutathione-s-transferase pi (GST-π), which is present in keratinocytes and melanocytes, has a protective role against tumor progression, specifically sun exposure-associated melanoma cells. This report makes enzyme GST-π possible to be used as an adjuvant marker of a chronically sun-damaged melanoma pathway [12]. A systematic review performed by Dulokthornsakul et al. also stated there was a trend that glutathione might lighten skin color at sun-exposed areas. However, its skin-whitening effect was still inconclusive, with its inconsistent findings from several studies [13].

The limitations of oral clinical trials are evident since the ability of GSH as an intact molecule decreases as it passes through the gastrointestinal tract. To pass the intestinal tract, glutathione has to be broken down into three amino acid components prior to absorption. Several animal studies showed that intact glutathione molecules may be absorbed through the small intestine, thus increasing plasma glutathione level. This would not be achieved if amino acid component is given in equivalent amount.

On the contrary, a study of 30 Filipino women with Fitzpatrick skin type IV or V used glutathione in oral mucosal preparations (lozenges). Handog et al. demonstrated that melanin index decreased significantly either in sun-exposed or sun-protected areas. Nevertheless, subjective assessments only showed mild to moderate skin-whitening effects [11].

These findings are applicable to Indonesian populace. All appraised studies were conducted on individuals with similar characteristics (i.e., healthy women). This is especially true since women tend to long for brighter skin color than men. In addition, this will exclude any different hormonal effects that may act as confounders. From risk-benefit analysis, oral glutathione consumption shows skin-whitening effects only in some areas. For cosmetic purposes, patients will have to bear their own costs as health insurances do not cover cosmetic expenses. Currently, systemic glutathione is yet to be legally distributed in Indonesia as its efficacy and safety profiles are still unknown.

Glutathione is not recommended for long-term use, especially if the patient shows noncompliance to maintenance therapies, including protection from UV rays and sunscreen use. Despite the fact that the appraised meta-analysis is valid and evident, in addition with the fact that appraised studies were conducted in Asia (Thailand and Philippines), clinical trials regarding the use of systemic glutathione in Indonesian populace need to be conducted.

## 7. Conclusion and Recommendations

Based on evidence-based critical appraisal of three trials, the role of systemic glutathione is not effective enough as a skin-whitening agent as it was only effective in some areas. Furthermore, skin color will return to its original state following withdrawal of glutathione consumption; hence, long-term effects are unsustainable. However, more researches need to be conducted in order to investigate how much time is needed for skin color to return to its original state following drug withdrawal.

## Figures and Tables

**Table 1:** Literature search strategy.

Database	Key words	Results	Included
Clinical Key	Oral glutathione as skin whitening	3	1
Cochrane	Glutathione	2	0
Journal of the American Academy of Dermatology	Glutathione in Article title AND skin in Article title	2	1
Taylor and Francis Online	Glutathione as skin whitening	1	1
PubMed	(((glutathione [Title]) OR glutathion [Title]) AND skin whitening[Title])	3	1
ScienceDirect	TITLE-ABSTR-KEY (glutathione) and TITLE-ABSTR-KEY (skin whitening)	5	1

**Table 2:** Summary of critical appraisal of included RCTs.

Author	Validity							Relevance			
	Study design	Subject size	Randomization	Intention to treat	Blinding	Equal treatment	Similar baseline characteristics	Domain	Determinant	Outcome measurements	Level of evidence
Arjinpathana and Asawanonda (2010) [1]	RCT	60	+	+	+	+	+	+	+	+	2
Zubair et al. (2016) [4]	RCT	32	—	—	—	+	+	+	+	+	2
Weschawalit et al. (2017) [3]	RCT	60	+	+	+	+	+	+	+	+	2

**Table 3:** Outcome of included trials.

Author	Objectives	Results	Summary
Arjinpathana and Asawanonda [1] (2012)	Comparison between baseline and posttreatment on melanin index measured by Mexameter (face, sun-exposed forearm, sun-protected arm) and VISIA™ CR system (UV spots, evenness, and pores) for each group as well as between groups.	*Baseline vs posttreatment.* (1) Melanin index in 6 areas decreased significantly in glutathione groups. On the contrary, in placebo arm, melanin index increased in facial area and decreased in the other areas. (2) No significant difference between baseline and post-treatment on VISIA analysis. There were significant increases for right and left UV spot in placebo arm (*p*=0.006 and *p*=0.012, respectively). *Comparison between glutathione and placebo.* Melanin index in glutathione arm decreased significantly compared to the control in all sun-exposed areas, especially right side of the face (*p*=0.021) and left forearm (*p*=0.036). UV spot in glutathione arm increased slightly. However, increased skin smoothness and decreased pore size were also observed in this group. Compared to placebo, these findings were not significant.	Glutathione was significant in most of the sun-exposed areas.

Zubair et al. [4] (2016)	Changes in skin color were observed after 12 injections of glutathione as measured by Taylor hyperpigmentation scale in 2 sun-protected body areas: (1) medial arm under the axilla, and (2) lateral upper thigh.	Six out of 16 (37.5%) subjects experienced significant improvements in glutathione group and 3 (18.75%) subjects in placebo group. Nonetheless, within 6 months after the last glutathione injection, gradual skin color improvements disappeared, leaving only 1 (6.2%) out of 16 subjects with skin color improvements. Both groups (glutathione and placebo) did not demonstrate significant difference in skin color (*p*=0.985 and *p*=0.998, respectively).	This study demonstrated poor outcomes of glutathione in terms of efficacy as well as cost-effectiveness.

Weschawalit et al. [3] (2017)	Changes in melanin index as measured by Mexameter in six body parts (sun-exposed and sun-protected). Other measured parameters including quantitative evaluation of UV spots, pores, and evenness measured by VISIA™ CR system, TEWL (transepidermal water loss) by Tewameter® TM300, water content by Corneometer® CM825, skin elasticity by Cutometer MPA580®, and skin wrinkles by Visioscan®.	On all subjects, melanin index and UV spot on all areas including the face and arm of GSSG and GSH groups tend to be lower than placebo group (*p* > 0.05). No significant difference was observed between GSSG and GSH groups. Subgroup analysis of subjects aged >40 years who received GSH (*N* = 7) showed lower melanin index than placebo arm (*N* = 10, *p*=0.031). Melanin index on sun-exposed area of the left forearm in GSH group was lower than placebo (*p*=0.057).	GSSG and GSH showed significant effects toward melanin index only in some areas of the skin and certain age groups, especially subjects with age >40 years on sun-exposed area of right forearm (*p*=0.031). GSH was effective to reduce facial wrinkles at least in some anatomical locations, as compared to placebo. Both glutathione preparations increased skin elasticity either in sun-exposed or sun-protected areas.
